# Neurospheres from rat adipose-derived stem cells could be induced into functional Schwann cell-like cells in vitro

**DOI:** 10.1186/1471-2202-9-21

**Published:** 2008-02-12

**Authors:** Yongfeng Xu, Zhengshan Liu, Lan Liu, Cuiping Zhao, Fu Xiong, Chang Zhou, Yong Li, Yanchang Shan, Funing Peng, Cheng Zhang

**Affiliations:** 1 Department of Neurology, the First Affiliated Hospital, Sun Yat-sen University, Guangzhou, P. R. China; 2 Center for Stem Cell Biology and Tissue Engineering of Sun Yat-sen University, Guangzhou, P.R. China; 3 Uveitis Center, Sun Yat-sen Ophthalmic Center, Sun Yat-sen University, Guangzhou, P.R. China; 4 Department of Medical Genetics, Southern Medical University, Guangzhou, P.R. China

## Abstract

**Background:**

Schwann cells (SC) which are myelin-forming cells in peripheral nervous system are very useful for the treatment of diseases of peripheral nervous system and central nervous system. However, it is difficult to obtain sufficient large number of SC for clinical use, so alternative cell systems are desired.

**Results:**

Using a procedure similar to the one used for propagation of neural stem cells, we could induce rat adipose-derived stem cells (ADSC) into floating neurospheres. In addition to being able to differentiate into neuronal- and glial-like cells, neurospheres could be induced to differentiate into SC-like cells. SC-like cells were bi- or tri-polar in shape and immunopositive for nestin and SC markers p75, GFAP and S-100, identical to genuine SC. We also found that SC-like cells could induce the differentiation of SH-SY5Y neuroblastoma cells efficiently, perhaps through secretion of soluble substances. We showed further that SC-like cells could form myelin structures with PC12 cell neurites in vitro.

**Conclusion:**

These findings indicated that ADSC could differentiate into SC-like cells in terms of morphology, phenotype and functional capacities. SC-like cells induced from ADSC may be useful for the treatment of neurological diseases.

## Background

Schwann cells (SC) play a central role in the regeneration of peripheral nerve, and are essential for peripheral nerve development [1]. It is recognized that SC can provide an option for the treatment of diseases of central nervous system (CNS), such as multiple sclerosis [2]. In CNS, SC transplantation can promote the re-growth of nerve fibres despite unfavorable environment [3]; SC can remyelinate demyelinated axons of CNS [4]. SC can clear debris by phagocytosis and break down devastated myelin [5], which can provide an important prerequisite for successful remyelination in demyelinating diseases of CNS [6]. However, it is difficult to obtain sufficient large number of SC for clinical use, so alternative cell systems are desired.

Bone marrow stromal cells (MSCs) can be obtained easily, can be expanded in culture conditions for autologous transplantation, and MSCs can transdifferentiate along a SC lineage in vitro [7] and in vivo [8]. So, MSCs may be one of alternative cell systems for SC. However, for clinical use, MSCs have presented problems: MSCs procurement procedures are painful and frequently require general or spinal anesthesia and may yield low number of MSCs upon harvest [9]. For these reasons, many researchers begin to investigate alternative sources for MSCs.

Adipose tissue, like bone marrow, is derived from embryonic mesoderm. Cells isolated from adipose tissue, termed adipose-derived stem cells (ADSC), are self-renewal and can differentiate along several mesenchymal tissue lineages, including adipocytes, osteoblasts, myocytes, chondrocytes, endothelial cells and cardiomyocytes [10,11]. ADSC may also be induced into neurospheres [12,13] and neuronal-like cells in vitro [14], and intracerebral transplantation of human ADSC can improve the neurological deficits after cerebral ischemia in rats [15]. Subcutaneous adipose tissue is abundant, readily accessible, and relatively expendable. Liposuction is a common surgical procedure and it is safe, and a large number of cells can be obtained with minimal risk [16]. ADSC may be an ideal alternative cell source for SC. However, it is not known up to now whether ADSC could be induced into SC.

In this study, we found that rat ADSC could be converted into neurospheres, and these neurospheres could be induced into SC-like cells. SC-like cells could induce the differentiation of SH-SY5Y neuroblastoma cells efficiently, and could form myelin structures with neuronal neurites.

## Results

### Rat ADSC characterization

**Figure 1:**
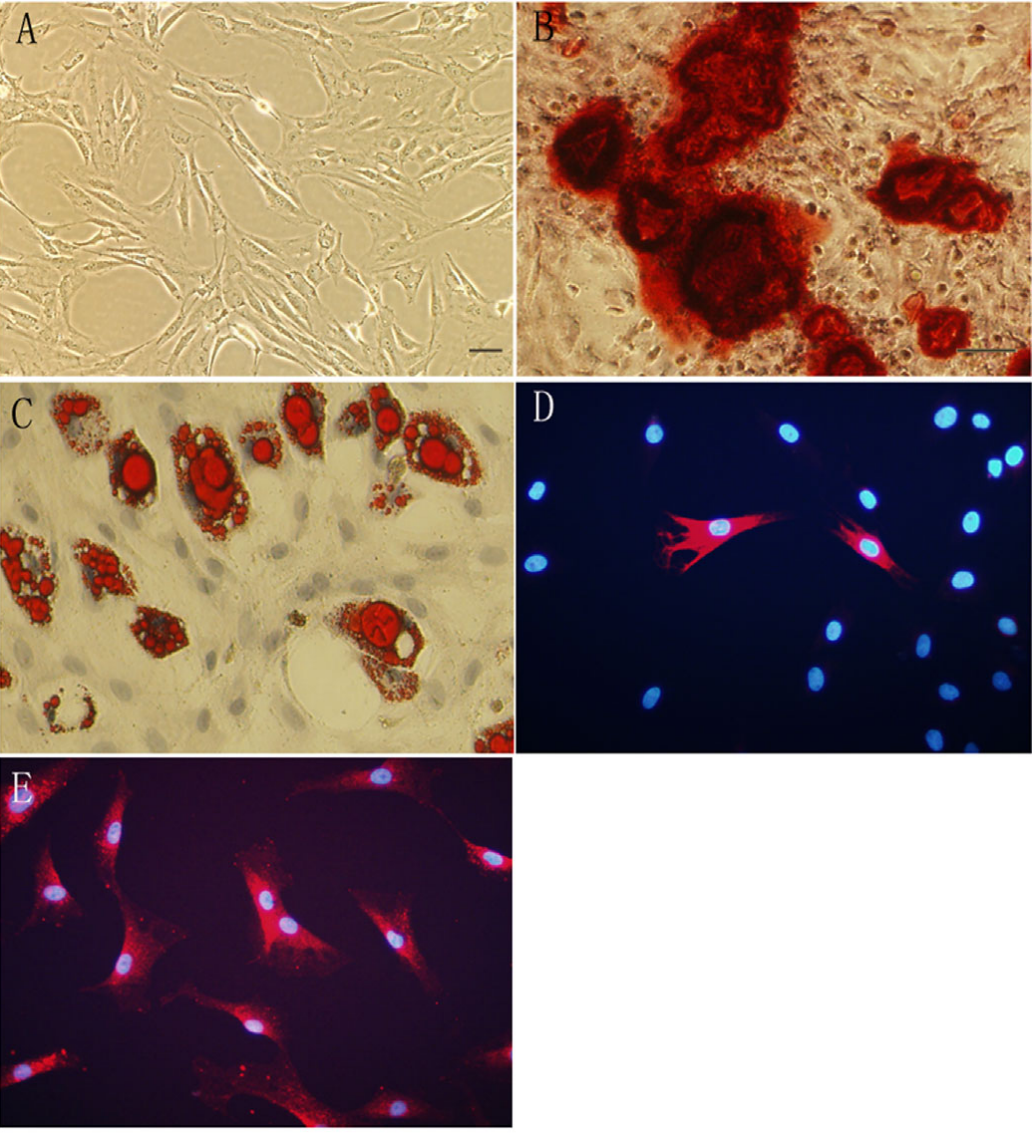
**Rat ADSC Characterization**. Rat ADSC were passaged for 3–5 times after initial plating of the primary culture. A: Under phase contrast, cultured rat ADSC are spindle-shaped. B, C: Rat ADSC could undergo multi-lineage differentiation, including osteogenesis (B) and adipogenesis (C), as visualized by staining with Alizarin Red S and Oil-Red O, respectively. D, E: Immunocytochemistry of rat ADSC within 3–5 passages. About 5 ± 3% of rat ADSC express neural stem cell marker nestin (D), whereas almost all of the rat ADSC express mesodermal marker fibronectin (E). Nuclei are labeled with DAPI (blue). Bar, A: 50 μm; B-E: 50 μm.

**Figure 2:**
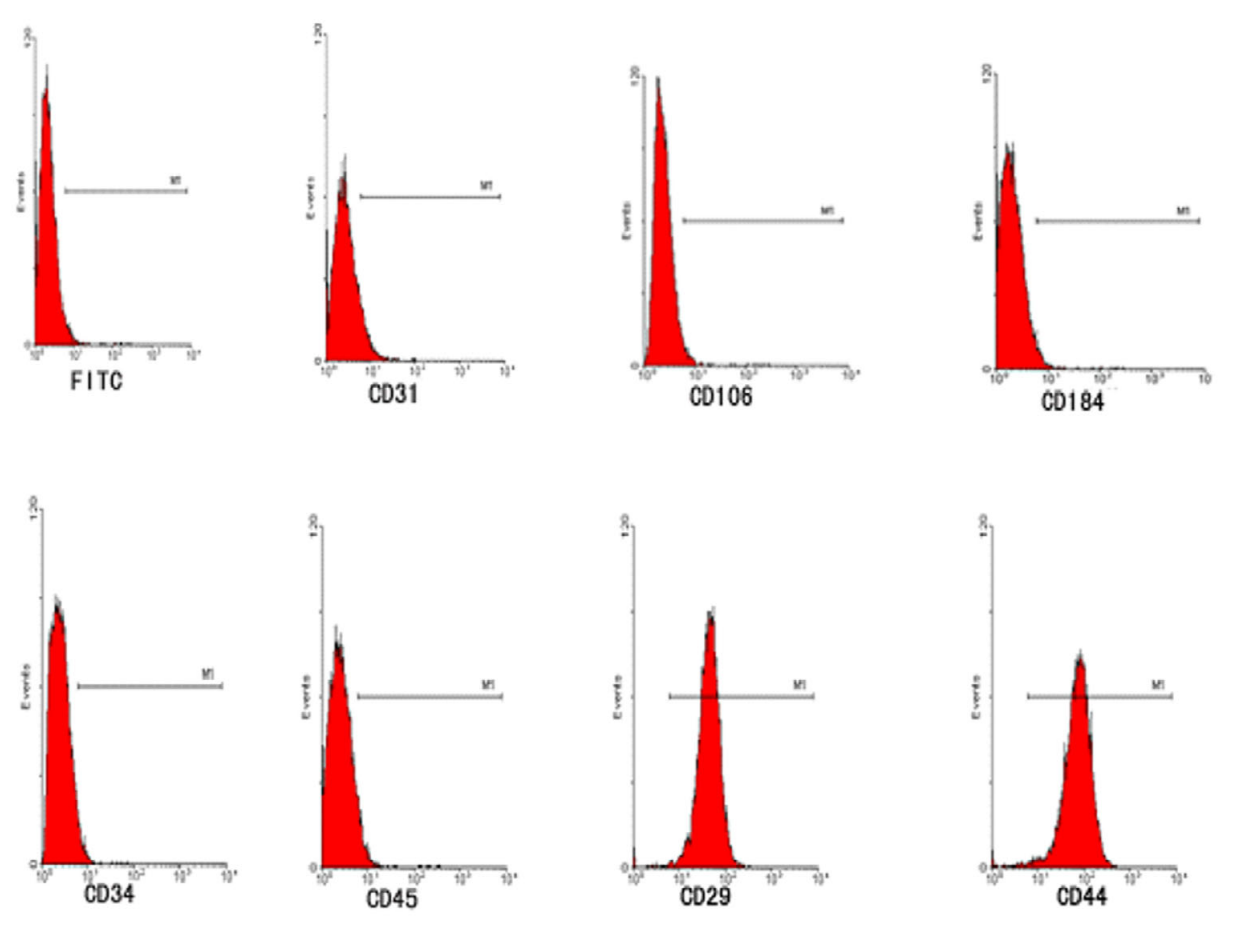
**Flow cytometric analysis of rat ADSC**. Rat ADSC within 3–5 passages were harvested and detected of specific cell surface antigens. Cells stained with a FITC-conjugated nonspecific IgG were examined as a control (FITC). Flow cytometric analysis shows that rat ADSC do not express CD31, CD106, CD184, CD34 and CD45, but express CD29 and CD44.

Within 3–5 passages after initial plating of the primary culture, rat ADSC appeared to be a mono-layer of large and flat cells (Figure 1A). Confluent rat ADSC showed a spindle-shaped, fibroblastic morphology. Rat ADSC could be passaged for at least 10 times, with a doubling time of 2.8 days. Flow cytometry analysis of rat ADSC within 3–5 passages showed that rat ADSC were CD29 and CD44 positive, but CD31, CD106, CD184, CD34 and CD45 negative (Figure 2). Rat ADSC did not spontaneously differentiate during culture expansion. When cultured in lineage-specific differentiation culture medium, rat ADSC within 3–5 passages could undergo osteogenic (Figure 1B) and adipogenic (Figure 1C) differentiation. About 5 ± 3% of rat ADSC within 3–5 passages were nestin positive (Figure 1D), whereas almost all of rat ADSC expressed mesodermal marker fibronectin (Figure 1E). Markers of neuronal or glial cell types, such as beta-tubulin III, GFAP, p75 and S-100 could not be detected in rat ADSC (not shown).

### Conversion of rat ADSC into neurospheres

**Figure 3:**
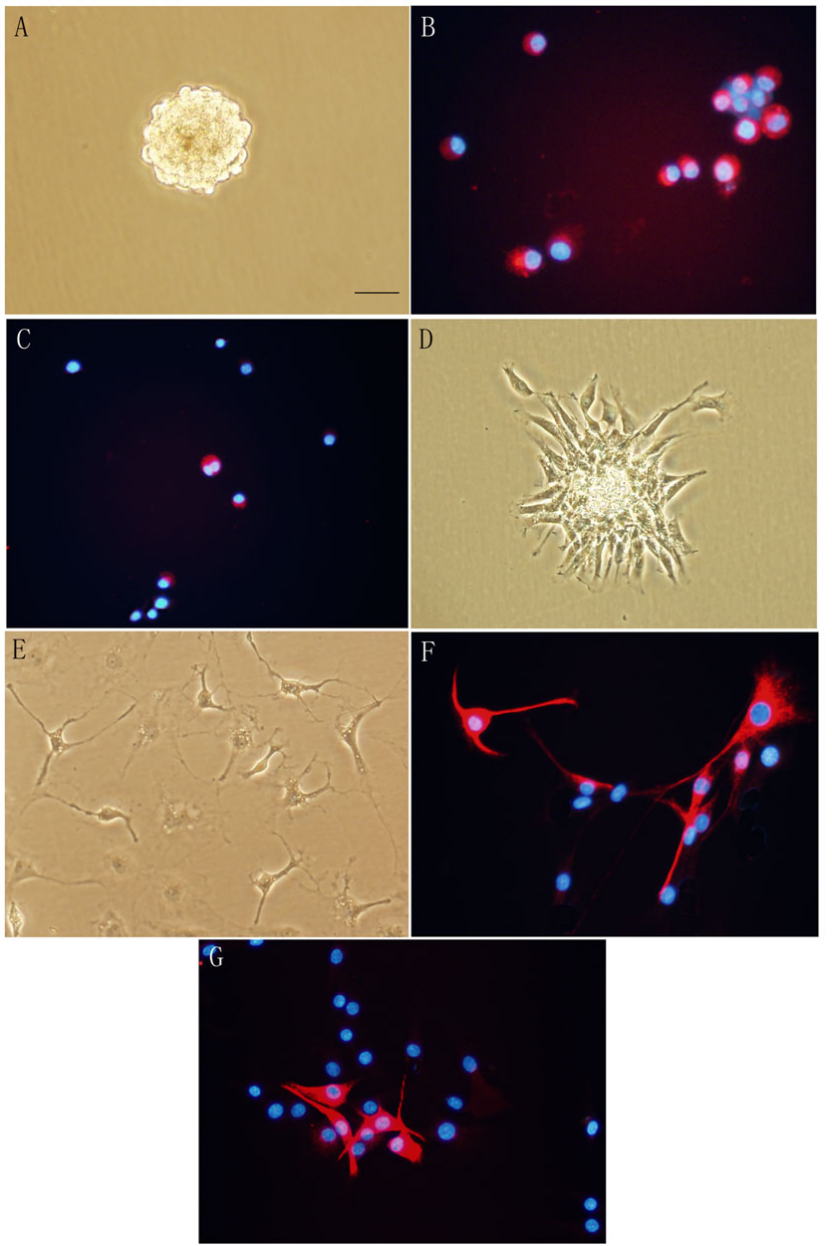
**Characterization of neurospheres converted from rat ADSC**. A: Under phase contrast, neurospheres grow as spheres of floating cells. B, C: Immunocytochemistry of neurospheres. Neurospheres were triturated before being examined. About 85 ± 7% of the neurosphere cells express nestin (B), whereas only a small part of neurosphere cells express fibronectin (C). D-G: Neurosphere cells can differentiate into neural cells. Neurospheres were cultured and maintained for 10 days in Neurobasal^® ^medium supplemented only with B27 supplement on poly-L-lysine-coated substrate. Neurospheres attach to the bottom of the culture dish and protrude cell processes (D), some cells gain a neuronal morphology and the processes grow much longer 10 days after plating (E); Immunocytochemistry shows that part of the differentiated neurosphere cells express neuronal marker beta-tubulin III (F) and glial marker GFAP (G) 10 days after plating. Nuclei are labeled with DAPI (blue). Bar, 50 μm.

ADSC can be converted into neurospheres using a procedure similar to the one used for the propagation of neural stem cells [12,13]. Rat ADSC within 3–5 passages were detached, and re-plated in serum-free DMEM/F12 medium supplemented with epidermal growth factor (EGF) and basic fribroblast growth factor (bFGF). Few cells adhered to the surface of flasks. A lot of small spheres of floating cells appeared after 2–4 days in conversion culture, and these spheres could proliferate in vitro for up to 2 months (Figure 3A). Neurospheres could be passaged every 7–10 days, with an estimated doubling time of 3 days. More than 90% of rat ADSC converted into neurospheres. About 85 ± 7% of neurosphere cells expressed nestin (Figure 3B), whereas only a small part of neurosphere cells expressed fibronectin (Figure 3C). The expression of beta-tubulin III, GFAP, S-100 and p75 were undetectable in neurosphere cells (not shown). As soon as the neurospheres were plated in poly-L-lysine-coated chamber slides in Neurobasal^® ^medium supplemented with only B27, the neurospheres began to spread across the growth surface (Figure 3D). Ten days after differentiation, a lot of differentiated neurosphere cells would gain a neuronal morphology, and the cell processes would grow much longer (Figure 3E). Immunostaining showed that 29 ± 6% and 22 ± 5% of the differentiated neurosphere cells were positive for neuronal marker beta-tubulin III (Figure 3F) and glial marker GFAP (Figure 3G), respectively.

### Neurospheres could be induced to differentiate along a SC lineage

**Figure 4:**
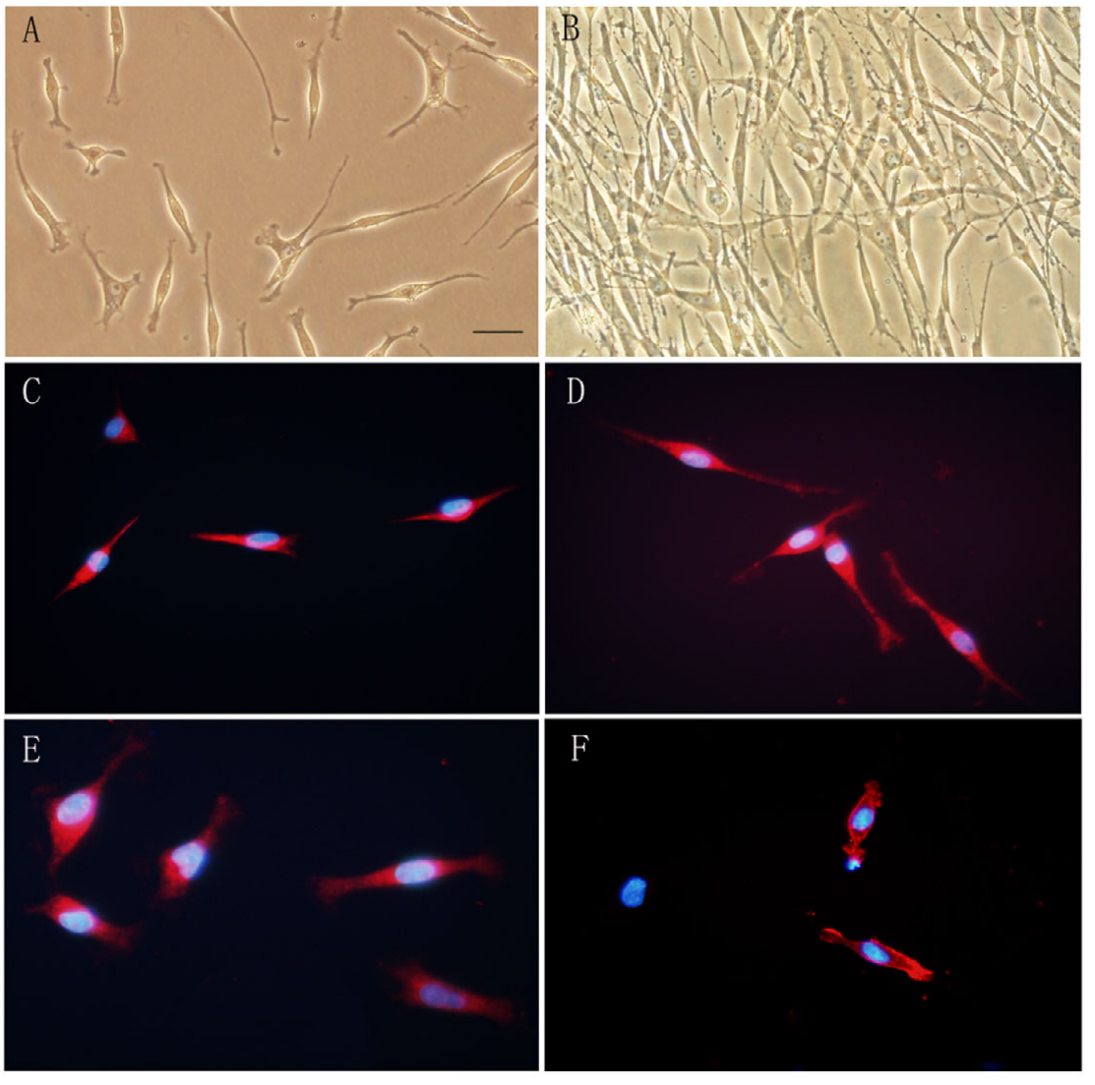
**Neurosphere cells could differentiate into SC-like cells**. Neurosphere cells were cultured for 48 hours on poly-L-lysine-coated six-well chamber slides in SC differentiation medium. A, B: Under phase contrast, most of the differentiated neurosphere cells (SC-like cells) are bi- or tri-polar, spindle-like 48 hours after differentiation (A), and these spindle-like cells can proliferate to a higher cell density when the SC differentiation medium was replaced with DMEM plus 10% FBS 48 hours after differentiation (B). C-F: Immunocytochemistry of SC-like cells. Almost all the SC-like cells express nestin (C), GFAP (D) and S-100 (E) in the cytoplasm, and 35 ± 5% of the SC-like cells express p75 in the cytomembrane (F). Nuclei are labeled with DAPI (blue). Bar, 50 μm.

Triturated neurospheres were re-plated in poly-L-lysine-coated six-well chamber slides in SC differentiation medium which contained all-trans-retinoic acid (RA), forskolin (FSK), platelet-derived growth factor (PDGF-BB) and Heregulin. Some bi-polar, spindle-like cells began to appear at 12 hours after differentiation, and almost all of cells were bi- or tri-polar, spindle-like at 24 and 48 hours after differentiation (Figure 4A), similar to the cultured immature genuine SC. About 60 hours after differentiation, a lot of differentiated neurosphere cells (i.e., SC-like cells) would aggregate and float, obviously dead. Forty-eight hours after differentiation, we replaced the SC differentiation medium with DMEM plus 10% FBS, then we observed that SC-like cells could survive, proliferate to a higher cell density (Figure 4B) and could be passaged at least for 1 to 2 times without changing their morphology and phenotype. Forty eight hours after differentiation, we detected the expression of nestin which is normally expressed in genuine SC [17] and SC markers p75, S-100 and GFAP [1,18] to evaluate the nature of SC-like cells. Immunostaining showed that almost all of the SC-like cells expressed nestin (Figure 4C), GFAP (Figure 4D) and S-100 (Figure 4E), and 35 ± 5% of the SC-like cells expressed p75 (Figure 4F).

### SC-like cells could induce the differentiation of SH-SY5Y cells efficiently

We used SH-SY5Y cells to evaluate whether SC-like cells could secrete soluble factors since genuine SC can induce the differentiation of SH-SY5Y neuroblastoma cells efficiently through production of soluble factors [19].

**Figure 5:**
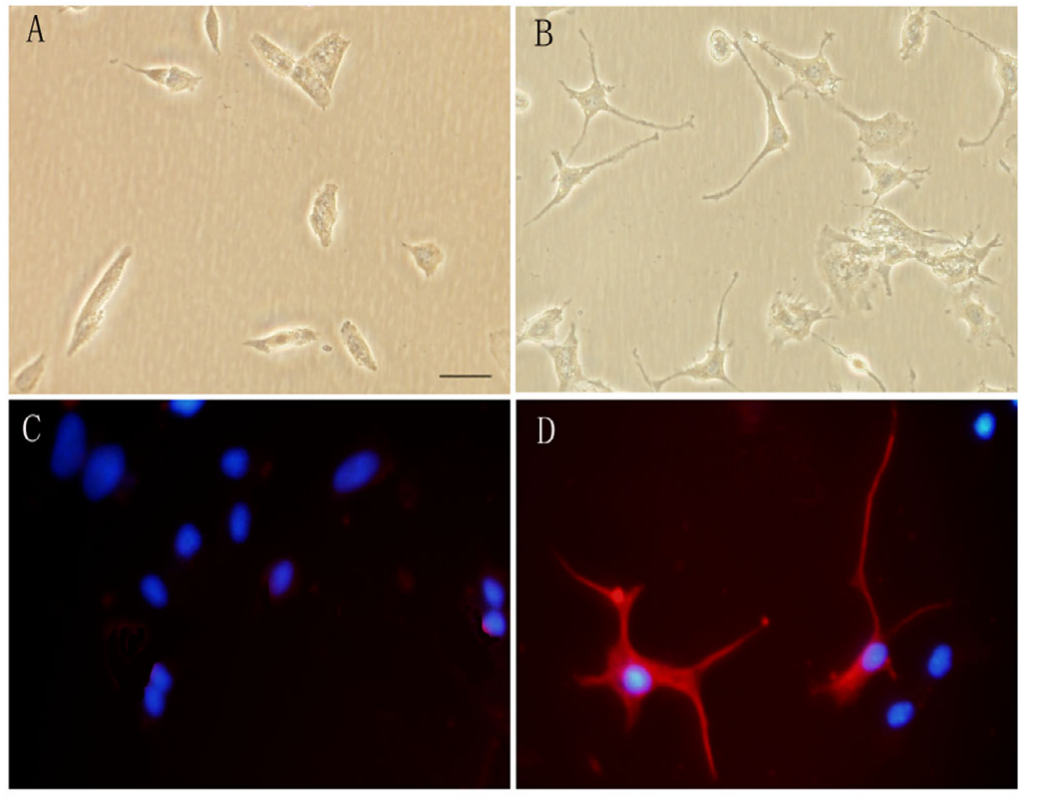
**SC-like cells can induce the differentiation of SH-SY5Y neuroblastoma cells**. SH-SY5Y cells were cultured in DMEM plus 2% FBS (control group) or SC-like cell-CM for 3 days. A, B: Under phase contrast, neurite outgrowth is minimal-to-no in control group (A), whereas many of the SH-SY5Y cells extend long neurites in SC-like cell-CM group (B). C and D: Immunocytochemistry of beta-tubulin III in control group and SC-like cell-CM group. Few or no SH-SY5Y cells are beta-tubulin III positive in control group (C), whereas 31 ± 6% of the SH-SY5Y cells are beta-tubulin III positive in SC-like cell-CM group (D). Nuclei are labeled with DAPI (blue). Bar, 50 μm.

After being cultured in the conditioned medium (CM) collected from SC-like cells for 3 days, part of SH-SY5Y cells showed neurite outgrowth under phase contrast (Figure 5B), whereas there was no neurite outgrowth in control group (Figure 5A). Immunofluorescent staining confirmed that 31 ± 6% of SH-SH5Y cells expressed neuronal marker beta-tubulin III after being cultured in CM from SC-like cells for 3 days (Figure 5D); in contrast, beta-tubulin III positive cells were very rare or no in control group (Figure 5C).

### SC-like cells could form myelin structures with neuronal neurites

**Figure 6:**
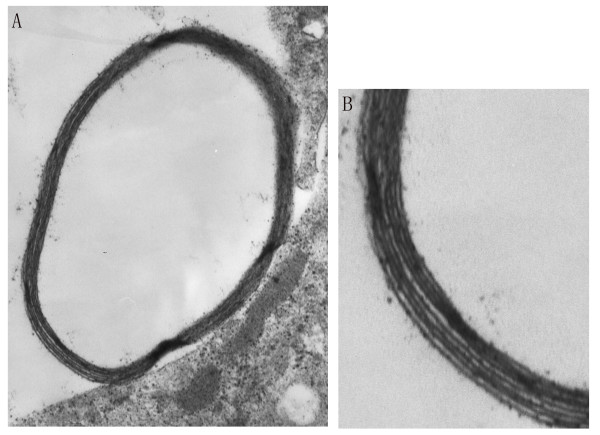
**SC-like cells could form myelin structures with neuronal neurites**. PC12 cells and SC-like cells from rat ADSC were co-cultured for 14 days. Electron micrographs show that compact myelin structures could be seen (A), and myelin structures are composed of multiple layers of membranes (B). A, ×21000; B, ×63000.

PC12 cells (rat pheochromocytoma cell line) were used to assess the myelinating capacity of SC-like cells since genuine SC can induce the differentiation of PC12 cells and form myelin structures with PC12 cell neurites [20]. PC12 cells had none or short processes. Most of PC12 cells stretched out noticeable processes after being cultured in DMEM plus 10% FBS for a few passages. Addition of SC-like cells induced a rapid neuronal-like differentiation of PC12 cells, and extension of neurites could be observed. Electron microscopy results showed that after 14 days, PC12/SC-like cell co-culture formed myelin structures, and a lot of myelin structures were compact (Figure 6A). The myelin structures were composed of multiple layers of membranes (Figure 6B). Rat ADSC could not form myelin structures with PC12 cell neurites (not shown).

## Discussion

Our results demonstrate that rat ADSC could be converted into neurospheres using a procedure similar to the one used for propagation of genuine neural stem cells. In addition to generating neuronal- and glial-like cells, neurosphere cells from rat ADSC could differentiate into SC-like cells. We showed further that SC-like cells were functional since these cells could secrete soluble factors and form myelin structures with neuronal neurites. Functional properties, especially formation of myelin structures with neuronal neurites, further indicated that SC-like cells from rat ADSC were closely similar to genuine SC.

Primary cultures of adipose tissue are heterogeneous, containing hepatopoietic cells, endothelial cells, smooth muscle cells and pericytes [9]. However, the number of these other cells is small, and the frequency of these other cells will diminish quickly through serial passages [21]. Also, ADSC can differentiate into several mesenchymal tissue lineages, including adipocyte and osteoblast. Rat ADSC within 3–5 passages we used in our experiment were CD29 and CD44 positive, CD31, CD106, CD184, CD34 and CD45 negative, and could undergo osteogenic and adipogenic differentiation. All these characteristics of rat ADSC in our experiments are consistent with previous reports [10].

Although ADSC and MSCs share many common biological characteristics, the two populations are not identical [12]. Immunocytochemical analysis shows that surface epitope profiles of the two populations are different [10]; although it is well established that both MSCs and ADSC can undergo chondrogenic differentiation [22], Kang et al show that ADSC but not MSCs could undergo chondrogenic differentiation under the conditions used in their study; ADSC may have significantly higher neural differentiation capacities than those of MSCs [12]; the distinctions between the two populations may also extend down to the gene level [10,12]. Although MSCs can be induced to differentiate along SC lineage [7], the differences between the two populations mentioned above suggest that whether ADSC could be induced to differentiate along SC lineage needs to be confirmed.

Some recent studies show that neural crest stem cells can be harvested by means of neurosphere method from various seemingly "mesodermal" tissues of adult animals, such as heart [23] and hair follicular dermal papilla [24]. Kang et al. show that ADSC can be converted into neurospheres [12], and a preliminary report further suggests that neurosphere cells derived from ADSC may have neural crest-like properties [13]. In our experiment, since neurospheres converted from rat ADSC could differentiate into SC-like cells which belong to peripheral nervous system, these neurospheres should have the characteristics of peripheral nervous system.

Recently, nestin expression has also been observed in myogenic cells, hepatic cells and endothelial cells, which indicates that nestin may not be used as a specific marker for neural stem cells. However, in our experiment, neurosphere cells from rat ADSC can be induced into neuronal- and glial-like cells, which strongly indicates that neurosphere cells derived from rat ADSC have neural stem cell-like properties. Hermann et al suggest that neural stem cell-like cells converted from MSCs are real neural stem cells [25]. The immature neural stem cells would be more suitable for the treatment of neurodegenerative diseases than fully differentiated neural cells, because fully differentiated neurons can not survive detachment and subsequent transplantation procedures [26].

Neural stem cells from CNS can be maintained in an undifferentiated status by bFGF and EGF [27,28]. When exposed to RA, neural stem cells will exit from cell cycle and differentiate into nerve cells [29]. In our experiment, neurosphere cells will differentiate in the presence of RA and in the absence of EGF and bFGF.

FSK can elevate the level of intracellular cyclic adenosine monophosphate (cAMP) and cAMP signal may be an intracellular signal during several different stages of SC development. In cultured SC, cAMP elevation can mimick SC responses in the presence of axons during myelination in vivo [30]. In addititon, FSK can enhance the responsiveness of SC to SC mitogens, such as PDGF-BB and glial growth factor [31]. PDGF-BB can induce SC proliferation in the presence of serum and FSK [32]. Heregulin is a subtype of neuregulin-1 and neuregulin-1 is now regarded as the pivotal signal that controls SC at every stage of the lineage [33]. Neuregulin-1 type II, also known as glial growth factor, can induce instructively cultured neural crest cells into SC [34]. In the presence of Heregulin (neuregulin-1 type I), MSCs can be induced into SC-like cells [35]. A mixture of cytokines mentioned above may synergize to induce neurosphere cells into SC-like cells.

SC can produce a number of neurotrophic factors, and a combination of these and other SC-derived soluble factors have been referred to as 'anti-neuroblastoma' agents [36]. Pigment epithelium-derived factor is now regarded as the key factor responsible for SC's ability to induce the differentiation of SH-SY5Y cells [19]. It is likely that SC-like cells from rat ADSC in our experiment produced at least some 'anti-neuroblastoma' agents produced by genuine SC since SC-like cells could induce the differentiation of SH-SY5Y cells efficiently. SC-like cells induced from MSCs can cause neurite growth of dorsal root ganglion neurons in vitro [7], which supports that SC-like cells from rat ADSC may produce some soluble factors. These SC-derived factors, such as pigment epithelium-derived factor, can promote survival and neurite outgrowth of neurons. SC-like cells from ADSC may be useful for the treatment of diseases in peripheral nervous system (e.g., nerve injuries) and CNS (e.g., multiple sclerosis).

## Conclusion

Our research indicated that ADSC could differentiate into SC-like cells in terms of morphology, phenotype and functional capacities. SC-like cells induced from ADSC may be useful for the treatment of neurological diseases.

## Methods

### Cell culture

The local ethics committee approved the animal experimentation protocols and all animal experiments were performed according to Sun Yat-sen university guidelines for animal care. Four- to 8- week-old, male, Sprague-Dawley rats were used for the isolation of rat ADSC. Animals were housed under standard conditions. After sacrifice of the rats, the inguinal fat pad was harvested, and rat ADSC were isolated using a published method [14]. Briefly, the adipose tissue was dissociated mechanically, digested using collagenase type I (Gibco, Carlsbad, CA, USA). The suspension was centrifuged to separate the floating adipocytes from the stromal vascular fraction. Then the cells in the stromal vascular fraction were cultured in Dulbecco's modified Eagle's medium (DMEM; Gibco) supplemented with 10% fetal bovine serum (FBS; Gibco). After 24 hours, the non-adherent cells were eliminated by changing the medium. Rat ADSC were passaged for 3–5 times before being used for the experiments.

SH-SY5Y neuroblastoma cell line and PC12 cells (rat pheochromocytoma cell line) were obtained from the American Tissue Type and Culture. SH-SY5Y cells were cultured in DMEM plus 10% FBS in 5%CO_2 _at 37°C. PC12 cells were cultured in DMEM/F12 (1:1, Gibco) supplemented with 15% horse serum (Gibco) and 2.5% FBS at 37°C in 5% CO_2_.

### Flow cytometry

Rat ADSC within 3–5 passages after the initial plating of the primary culture were harvested by trypsinization, then the cells were fixed in neutralized 2% paraformaldehyde solution for 30 minutes. The fixed cells were washed twice with PBS and incubated with antibodies to the following antigens: CD31, CD106, CD184, CD34, CD45, CD29 and CD44 (all from Chemicon, Temecula, CA, USA) for 30 minutes. Primary antibodies were directly conjugated with FITC. For isotype control, nonspecific FITC-conjugated IgG was substituted for the primary antibodies [10]. Flow cytometry was performed with a FACscan flow cytometer (Becton Dickinson, San Jose, CA).

### Adipogenic and osteogenic differentiation of rat ADSC

Rat ADSC within 3–5 passages were used to verify the multi-potential differentiation capacity. Cells were grown to at least 80% confluence before being cultured in the induction medium. To induce osteogenic differentiation, rat ADSC were cultured for three weeks in DMEM supplemented with 10% FBS, 0.1 μM dexamethasone, 50 μM ascorbate-2-phosphate, 10 mM beta-glycerophosphate. Mineralization of the extracellular matrix was visualized by staining with Alizarin Red S. To induce adipogenic differentiation, rat ADSC were cultured for three weeks in DMEM supplemented with 10% FBS, 0.5 mM isobutyl-methylxanthine (IBMX), 1 μM dexamethasone, 10 μM insulin, 200 μM indomethacin. Adipogenic differentiation was confirmed by staining with Oil-Red O.

### Induction of rat ADSC into neurospheres

Rat ADSC within 3–5 passages were induced into neurospheres. In detail, we dissociated rat ADSC (80–90% confluence) with 0.25% trypsin (Gibco) and then plated them on culture flasks at a concentration of 1–2 × 10^5^/cm^2 ^in DMEM/F12 (1:1) supplemented with 20 ng/ml EGF (Peprotech, London, UK), 20 ng/ml bFGF (Peprotech) and B27 (1:50, Gibco) (neurosphere growth medium, NG medium) at 37°C in 5%CO_2 _[12,13]. We added fresh NG medium every 3 to 4 days and changed the medium once a week. Neurospheres were passaged every 7 to 10 days by being triturated using a fire-polished Pasteur pipette and being re-plated in fresh medium. We triturated neurospheres and re-plated them in poly-L-lysine (Sigma, St Louis, MO, USA)-coated six-well chamber slides for terminal differentiation experiments.

### Terminal differentiation of neurospheres

To induce neurospheres into neural cells, neruospheres from rat ADSC were plated in poly-L-lysine-coated six-well chamber slides and cultured in Neurobasal^® ^medium (Gibco) supplemented with B27 (1:50) for 10 days. During differentiation, 70% of the medium was replaced every 4 days [12].

To induce neurospheres into SC-like cells, we triturated neurospheres and re-plated them in poly-L-lysine-coated six-well chamber slides at a density of 2.0–2.5 × 10^5 ^cells/cm^2^. We cultured the cells in NG medium for 6 to 8 hours first, then we removed the NG medium, and washed the cells twice with phosphate buffered saline (PBS). Then the cells were induced to differentiate for 48 hours in DMEM supplemented with 10% FBS, 0.5 μM RA (Sigma), 5 μM FSK (Alexis, Lausen, Switzerland), 10 ng/ml PDGF-BB (Peprotech) and 200 ng/ml Heregulin-beta1 (Peprotech) (SC differentiation medium). In some experiments, the SC differentiation medium was replaced with DMEM plus 10% FBS 48 hours after differentiation.

### CM preparation

CM was collected from SC-like cells. SC-like cells were grown to 80% confluence. We aspirated the medium and rinsed the cells twice with 5 ml of PBS. We then aspirated the rinse medium and added 4 ml of DMEM supplemented with 2% FBS. The cells were cultured at 37°C in 5% CO_2_. Forty-eight hours later, we harvested and centrifuged the medium (1000 g, 5 minutes), and collected the supernatant as SC-like cell-CM [19].

### Assessment of the differentiation of SH-SY5Y cells

We dissociated SH-SY5Y cells, and seeded 1 ml of cell suspensions containing 1.25 × 10^4 ^cells/ml in each well of 24-well plates coated with poly-L-lysine. Twenty-four hours later, we washed SH-SY5Y cells twice with PBS and cultured the cells for 3 days in the following medium: 1) DMEM with 2% FBS (control group); 2) SC-like cell-CM (SC-like cell-CM group). A cell whose neurite length was longer than 50 μm was regarded as differentiated [19]. We used antibody against beta-tubulin III protein (Chemicon) to confirm the neuronal differentiation of SH-SY5Y cells.

### Immunocytochemistry

We detected the expression of each antigen for 2 to 4 times in independent experiments. We fixed the cells with 4% paraformaldehyde, blocked the cells with normal goat serum. Then anti-nestin (mouse monoclonal, 1:40), anti-beta-tubulin III (mouse monoclonal, 1:100), anti-glial filament acidic protein (GFAP; mouse monoclonal, 1:400), anti-S-100 (mouse monoclonal, 1:100), anti-p75 nerve growth factor receptor (p75; mouse monoclonal, 1:222) (all from Chemicon) and anti-fibronectin (mouse monoclonal, 1:250; Neomarkers, Fremont, CA, USA) were added. The primary antibodies were incubated overnight at 4°C. We used Cy3-conjugated goat anti-mouse antibody (Chemicon) as secondary antibody which was incubated at room temperature for 1 hour. Then we used DAPI (Sigma) to label the nuclei. Primary antibodies were omitted for control. We examined the cells with a fluorescence microscope (Olympus DP70, Japan).

### In vitro myelination assay

Before being used for the co-culture experiment, PC12 cells were cultured in DMEM plus 10% FBS for a few passages until most of PC12 cells stretched out noticeable processes [20]. PC12 cells cultured in DMEM plus 10% FBS for a few passages were dissociated and re-plated at a density of 500 cells/cm^2 ^in poly-L-lysine coated culture dishes in DMEM plus 10% FBS. After 12–24 hours, the medium was removed from PC12 cells, and 500 dissociated rat ADSC or 500 SC-like cells from rat ADSC were seeded into each dish, respectively. PC12/SC-like cells and PC12/rat ADSC were cultured in DMEM plus 10% FBS for 14 days, and the medium was changed every 2–3 days [20]. After 14 days, the cocultures were fixed in 2% glutaraldehyde in sodium cacodylate buffer at 4°C. Following treatment with 1% osmium tetroxide and 1% uranyl acetate, samples were embedded in epon. Ultra-thin sections (50–70 nm) were cut and mounted on Formvar-coated slot grids, and stained for 20 s in 1:1 supersaturated uranyl acetate in acetone followed by staining in 0.2% lead citrate. For examination a CM10 transmission electron microscope (Philips, Netherlands) was used. Electron microscopy was performed at electron microscopy center of Sun Yat-sen university.

### Statistical analysis

We photographed ten random fields per marker. We counted the number of positively stained cells and the total cell number as indicated by DAPI nuclear labeling, respectively. Data are expressed as means ± S.D for all samples. We used SPSS 11.0 to analyze the data. Statistical comparisons were made by student t test. We set statistical significance at p < 0.05 for all the tests performed.

## Abbreviations

SC: Schwann cells; ADSC: adipose-derived stem cells; MSCs: bone marrow stromal cells; CNS: central nervous system; CM: conditioned medium

## Authors' contributions

Yongfeng Xu and Zhengshan Liu: made substantial contributions to conception and design, especially Yongfeng Xu; carried out the experiments and analyzed the data; Yongfeng Xu drafted the manuscript.

Cheng Zhang: made contributions to conception and design, analyzed the data, revised it critically for important intellectual content.

Lan Liu, Cuiping Zhao, Fu Xiong, Chang Zhou, Yong Li, Yanchang Shan, Funing Peng: carried out some experiments and analysis.

All authors read and approved the final manuscript.
